# Intrauterine hCG application increases expression of endothelial cell–cell adhesion molecules in human

**DOI:** 10.1007/s00404-021-06031-9

**Published:** 2021-04-26

**Authors:** Michaela Bienert, Pardes Habib, Volker Buck, Irmgard Classen-Linke, Roman Skoblo, Benjamin Rösing

**Affiliations:** 1 Institute of Molecular and Cellular Anatomy, RWTH Aachen University, Wendlingweg 2, 52074 Aachen, Germany; 2 IFLB, Institute for Laboratory Medicine, Windscheidstr. 18, 10627 Berlin, Germany; 3 Department of Neurology, Medical Faculty, RWTH Aachen University, Pauwelsstrasse 30, 52074 Aachen, Germany; 4 Clinic for Gynecological Endocrinology and Reproductive Medicine, Medical Faculty, RWTH Aachen University, Pauwelsstrasse 30, 52074 Aachen, Germany

**Keywords:** Embryo implantation, HCG, ART, Window of implantation, Reproduction, Vascularization, Mesenchymal stroma cells

## Abstract

Endometrial receptivity is a decisive factor in human reproduction. Human chorionic gonadotropin (hCG) is one of the first embryonic signals that precedes the implantation by trophoblast invasion into the endometrium. Meta-analysis of randomized controlled trials reports a moderate-quality evidence for improved live birth rate for an intrauterine hCG dose ≥ 500 IU. Nevertheless, all hCG endometrial effects are not completely understood. We, therefore, utilized endometrial tissue from 12 patients after estradiol and progesterone treatment with or without intrauterine hCG flushing at the window of implantation (WOI) to analyze cellular composition by measuring marker proteins for stromal, endothelial, epithelial and immune cells. Flow cytometry analysis revealed that significantly more cells expressed the endothelial adhesion molecules VE-cadherin (CD144) and S-Endo-1 (CD146) after intrauterine hCG administration. In contrast, the endothelial marker CD31 and markers involved in vessel formation (VEGFR1 and VEGFR2) remained unchanged in their expression. Similarly, stroma markers (CD73, CD90 and CD105), epithelial markers (Desmocollin-2 and E-Cadherin) and immune cell markers (CD11b, CD45, CD79a and HLA-DR) displayed no alterations in their expression. This finding directs the focus on endothelial adhesion molecules as a potential mechanistically explanation of hCG conveyed increase of embryo implantation and pregnancy rates in women undergoing ART.

## Introduction

Despite major advances in assisted reproductive technologies (ART), embryo implantation is still one of the major hurdles for successful in vitro fertilization and intracytoplasmic sperm injection (IVF/ICSI) treatment [1]. Embryo implantation is a highly complex process at the cellular and molecular level involving attachment of blastocysts, which have removed their zona pellucida, to the maternal endometrium and subsequent invasion. In addition to the development and maturation of the pre-implantation embryo and the contact between embryo and endometrium, the hormonal preparation of the endometrium is of paramount importance for embryo implantation [2]. At the moment there is no standard therapy in clinical practice to guarantee successful implantation [3]. Therefore, new mechanisms that make the endometrium more receptive need to be investigated.

Meta-analysis port a moderate-quality evidence for improved live birth rate in cleavage-stage transfer for an intrauterine hCG dose ≥ 500 IU. Randomized controlled trials analyzed in meta-analysis are very heterogeneous and vary greatly in selection of patients recruited, number and stage of embryos transferred (cleavage or blastocyst stage), transfer in stimulated cycles, dosage and preparation of hCG, time and method of hCG application as well as evaluation of clinical results [4] Under physiological conditions hCG is expressed before implantation by the blastocyst and after implantation increasingly by the syncytiotrophoblast [5, 6]. In addition, hCG affects not only epithelial and stromal cells during decidualization [7] but also the endometrial vasculature [8]. Vascular growth is strongly induced by the well-known cytokine VEGF. Local infusion of hCG using an intrauterine micro-dialysis device during the luteal phase significantly stimulated VEGF expression [9].

The endometrium encompasses multiple cellular elements. Tubular glands are connected to the single-layered surface epithelium and are embedded into the stroma which consists of cell-rich, low-fiber connective tissue with immune cells migrating into during decidualization. The endometrium is divided into the functionalis, which is shed during desquamation, and the basalis from which the functionalis is cyclically regenerated [10]. Single-cell RNAseq of endometrial tissue samples identified stromal, immune (macrophages and lymphocytes), epithelial and endothelial cell clusters as the main cellular populations in the endometrium [11].

Cells isolated from the human endometrium, showed multipotent characteristics as they differentiated into smooth muscle cells, adipocytes, chondrocytes and osteoblasts and expressed the mesenchymal stem/stromal cell (MSC) markers CD73, CD90 and CD105 [12]. Identification of adult stem cells in the endometrium suggests that MSC have a key role during cyclic endometrial regeneration [13]. Due to the lack of a universally accepted surface marker, a set of characteristics was established by the International Society for Cellular Therapy (ISCT) to describe MSC [14]. Thus, a cell population is defined as an MSC population if CD73, CD90 and CD105 are expressed and CD11b, CD34, CD45 and CD79a are not.

The histological and functional subdivision of the endometrium suggests that the interplay of the different cell populations has an important role during implantation. In this work, the ISCT marker set is investigated in a stroma and stem cell cluster by quantification of CD34, CD73, CD90 and CD105 and in an immune cell cluster by quantification of CD11b, CD45 and CD79a at the protein level. To investigate the effects of hCG on the vasculature CD31, CD144, CD146, VEGFR1 and VEGFR2 were characterized within an endothelial cell cluster. Epithelial markers were investigated by determining E-Cadherin and Desmocollin-2 protein levels in an epithelial cell cluster. Epithelial cells are in directed contact with the stromal cells and play an important role during early embryo implantation. Epithelial integrity is generated by tight junctions, which are made of epithelial-cadherins like E-cadherin [15] which is important during early embryo implantation. Desmosomes are strong types of cell-to-cell adhesion types [16].

The explicit mechanism of action of hCG on the human endometrium is not fully understood. In this work, we investigate whether proteins of the stroma/stem cell, immune cell, endothelial or epithelial cell types are more likely to be altered by hCG in comparison to non-treated tissue.

## Materials and methods

### Subjects, hormonal pre-treatment and biopsy collection procedure

Endometrial samples were obtained in a diagnostic pipelle (Gynetics, endometrial curette #4164 Probet) procedure from twelve infertile women during their ART treatment. Endometrial tissue samples with intrauterine hCG administration (6 women in the HRT + hCG group) were compared to samples without hCG (6 women in the HRT group), to determine in vivo hCG effects on human endometrium. Endometrial sampling was performed in a clinical cohort in a diagnostic treatment cycle after previous fresh and frozen embryo transfers. Demographical and clinical characteristics of the cohort are presented in Table 1. The endometrial preparation for pipelle sampling followed the clinical routine procedure for a frozen embryo transfer and was performed as follows: Micronized estradiol (oral, 1–4 mg/days, Estrifam, Novo Nordisk Pharma, Germany) was given for 9–16 days until the endometrial diameter reached > 8 mm. All endometria had a sonographical trilaminar structure, and endogeneous progesterone was < 1.5 ng/ml before initiation of vaginal progesterone (400 mg/days for 137 h) application at 5 pm. All endometrial samples were from the fundal endometrial area after measuring the length of the uterine cavity (procedure in analogy to embryo transfer). All samples were taken by BR. Subjects in the HRT + hCG-group had an intrauterine flushing of 1000 IU hCG (urinary hCG, Brevactid, Ferring) in 200 µl NaCl solution via an embryo-transfer catheter (Cook Guardia). Intrauterine hCG application was scheduled at day 4 of vaginal progesterone at the time of potential cleavage stage embryo transfer in a frozen embryo transfer cycle. Endometrial sampling was done 137 h (d 5.7) after first vaginal progesterone at 10 am, representative for the estimated time of hatching and first trophoblast-endometrium contact in a comparable real embryo transfer cycle. The tissue was processed for further analysis within 90 min after sampling.

**Table 1:** Patient characteristics and clinical medical history

	HRT	HRT	HRT	HRT	HRT	HRT	HRT	HRT + hCG	HRT + hCG	HRT + hCG	HRT + hCG	HRT + hCG	HRT + hCG	HRT + hCG	*p *value
Patient ID	*#1*	*#2*	*#3*	*#4*	*#5*	*#6*	Ø ± SD	*#7*	*#8*	*#9*	*#10*	*#11*	*#12*	Ø ± SD	HRT vs HRT + hCG
Age [years]	32.00	31.00	28.00	33.00	26.00	41.00	32.0 ± 4.8	31.00	36.00	32.00	31.00	35.00	37.00	33.7 ± 2.7	*p* = 0.4674; ns
BMI	24.80	24.30	18.80	26.60	46.70	20.40	26.9 ± 10.1	18.30	19.40	25.30	29.40	19.80	23.10	22.5 ± 4.3	*p* = 0.4848; ns
HOMA	1.70	2.10	2.10	1.80	2.10	2.40	2.0 ± 0.3	1.80	1.60	2.40	2.80	1.90	1.50	2.0 ± 0.5	*p* = 0.6580; ns
Infertility time [month]	15.00	17.00	23.00	17.00	9.00	16.00	16.2 ± 4.5	25.00	14.00	19.00	19.00	21.00	8.00	17.7 ± 5.9	*p* = 0.5736; ns
Priors ETs [fresh, frozen]	1.00	1.00	2.00	3.00	1.00	3.00	1.8 ± 0.9	1.00	2.00	2.00	4.00	3.00	2.00	2.3 ± 1.0	*p* = 0.4199; ns
Cumulative ETs	2.00	1.00	4.00	6.00	2.00	6.00	3.5 ± 2.1	2.00	4.00	3.00	8.00	6.00	3.00	4.3 ± 2.3	*p* = 0.5736; ns
ART treatment	ICSI	ICSI	ICSI	ICSI	ICSI	ICSI		ICSI	ICSI	ICSI	ICSI	ICSI	ICSI		
Infertility type	Primary	Primary	Primary	Primary	Primary	Primary		Primary	Primary	Primary	Secondary	Primary	Secondary		
Gravity	0.00	0.00	0.00	0.00	0.00	0.00		0.00	0.00	0.00	1.00	0.00	1.00		
Parity	0.00	0.00	0.00	0.00	0.00	0.00		0.00	0.00	0.00	0.00	0.00	0.00		
Abortion	0.00	0.00	0.00	0.00	0.00	0.00		0.00	0.00	0.00	1.00	0.00	1.00		
Myome	0.00	0.00	0.00	0.00	0.00	0.00		0.00	0.00	0.00	0.00	0.00	Enucleation of intramural myoma in 2015^a^		
Endometriosis [wod, 0, ARSM I-V]^b^	0.00	ASRMI^c^	Wod	Wod	Wod	Wod		Wod	0.00	Wod	Wod	0.00	0.00		
Comorbidity	None	None	Epilepsy	None	Diabetis mellitus type 2 (HbA1c6,2)^d^	None		None	Asthma	Hashimoto-thyroidtis	None	None	Gilbert syndrome		
Medication	L-Thyroxine 50	None	Lamotrigine	None	Metformin	None		None	None	l-Thyroxine 75	None	None	none		

Females were treated with hormonal replacement therapy with (HRT + HCG) or without HCG (HRT) before biospsies collection

The target values are the average age in years, the average body mass index (BMI), homeostasis model assessment (HOMA), infertility time in month, prior embryo transfers (ETs) as fresh and frozen and cumulative ETs

HOMA index was calculated by insulin (μU/l) × (glucose (mmol/l)/22.5). Furthermore, ART treatment, infertility type, gravidity, parity, abortion, myoma, endometriosis status, comorbidities and respective medication are presented

Mean (Ø) and standard deviation (SD) are given. Significant between HRT and HRT + hCG group were assessed using nonparametric Mann–Whitney *U *test (*ns *not significant, confidence interval 95%)

^a^Samples used in this study were collected in 2019 and 2020

^b^Wod = without dysmenorrhea, 0 = negative laparoscopy/histology

^c^ASRM I (American Society for Reproductive Medicine II): diagnosed in laparoscopy in 2013, clinically inapparent since then

^d^Diabetis mellitus type 2 sufficently controlled controlled to HbA1c 6.2 [%]

Due to an effort to identify any uterine or endometrial factor for infertility, all patients had a 3-D saline hysterosonography or an office hysteroscopy as a part of our clinical diagnostic routine before initiation of ART treatment. Thus any structural uterine pathology was ruled out before endometrial testing.

### Ethics

The study was approved by the institutional ethics committee of the Medical Faculty of the University of Aachen (EK 201/14 and EK 074/16). Written informed consent was obtained from all patients.

### Cell isolation from human endometrium

Human endometrial tissue pieces weighing between 0.2 and 0.4 g were minced and incubated for 1 h at 37 °C with collagenase (1 mg/ml; Sigma, Germany) in DMEM/F12 without phenol red (Sigma, Germany). After centrifugation for 5 min at 100×*g*, the pellet was resuspended in PBS (Sigma) containing 0.1% steroid hormone-free fetal calf serum (FCS) (CC-Pro, CC-Pro GmbH, Germany). Afterwards the single-cell suspension was analyzed by flow cytometry.

### Flow cytometry

Cells isolated from the biopsies were directly resuspended in 100 µl PBS + 0.1% steroid hormone-free FCS (CC-Pro). Specific antibodies or isotype controls were added to the samples at concentrations recommended by the manufacturers and were incubated at 4 °C for 30 min. Isotype controls were APC mouse IgG1k, PE mouse IgG1k, FITC mouse IgG1k, PerCP-Cy5.5 mouse IgG1k and PerCP-Cy™5.5 mouse IgG2aκ (all BD Pharming). Antibodies for the stroma and immune cell cluster were PerCP-Cy5.5 mouse anti-human CD105, FITC mouse anti-human CD90, FITC mouse anti-human CD45, APC mouse anti-human CD79a, PE mouse anti-human CD11b, PerCP-Cy5. 5 mouse anti-human HLA-DR, PE mouse anti-human CD34 and APC mouse anti-human CD73 (all BD Pharming). Antibodies for the endothelial cell cluster were FITC mouse anti-human CD31, APC mouse anti-human CD309 VEGFR2 (both Miltenyi Biotech), PE mouse anti-human VEGFR1 (antibodiesonline), PE mouse anti-human CD144 (Thermo Fisher Scientific) and PE mouse anti-human CD146 (BD Bioscience). Antibodies for the epithelial cell cluster were mouse anti-human E-Cadherin as primary antibody (Origene) with the secondary antibody FITC goat anti-mouse (BD Bioscience) and rabbit anti-human Desmocollin 2 as the primary antibody (Progen) with the secondary antibody AF488 goat anti-rabbit (Thermo Fisher). After the staining cells were resuspended in 100 µl PBS + 0.1% FCS and subsequently centrifuged for 3 min at 200×*g*. Supernatant was removed and cells were resuspended in fresh PBS + 0.1% FCS. Washing was repeated 3 times before the cell suspension was transferred to FACS tubes. 20,000 events per sample were measured in a FACSCanto™ II flow cytometer (Becton Dickinson). Compensation was determined using the CompBeads set anti-mouse Ig, κ (BD Bioscience).

### Statistics

Data analysis and visualization were performed using GraphPad Prism (version 8.4.3, San Diego, CA, USA) and SPSS (IBM, NY, USA). Statistical analysis of the patient characteristics as well as different CD marker expression in the HRT and HRT + hCG group were analyzed by non-parametric unpaired Mann–Whitney *U* test. Data are given as arithmetic means ± SD. The confidence interval was set to 95%. Asterisks indicate significance between groups. If the expression of a CD marker in the HRT group differed significantly from the HRT + hCG group, the results were analyzed by multiple linear regression using SPSS software (IBM, NY, USA).

## Results

**Fig. 1:**
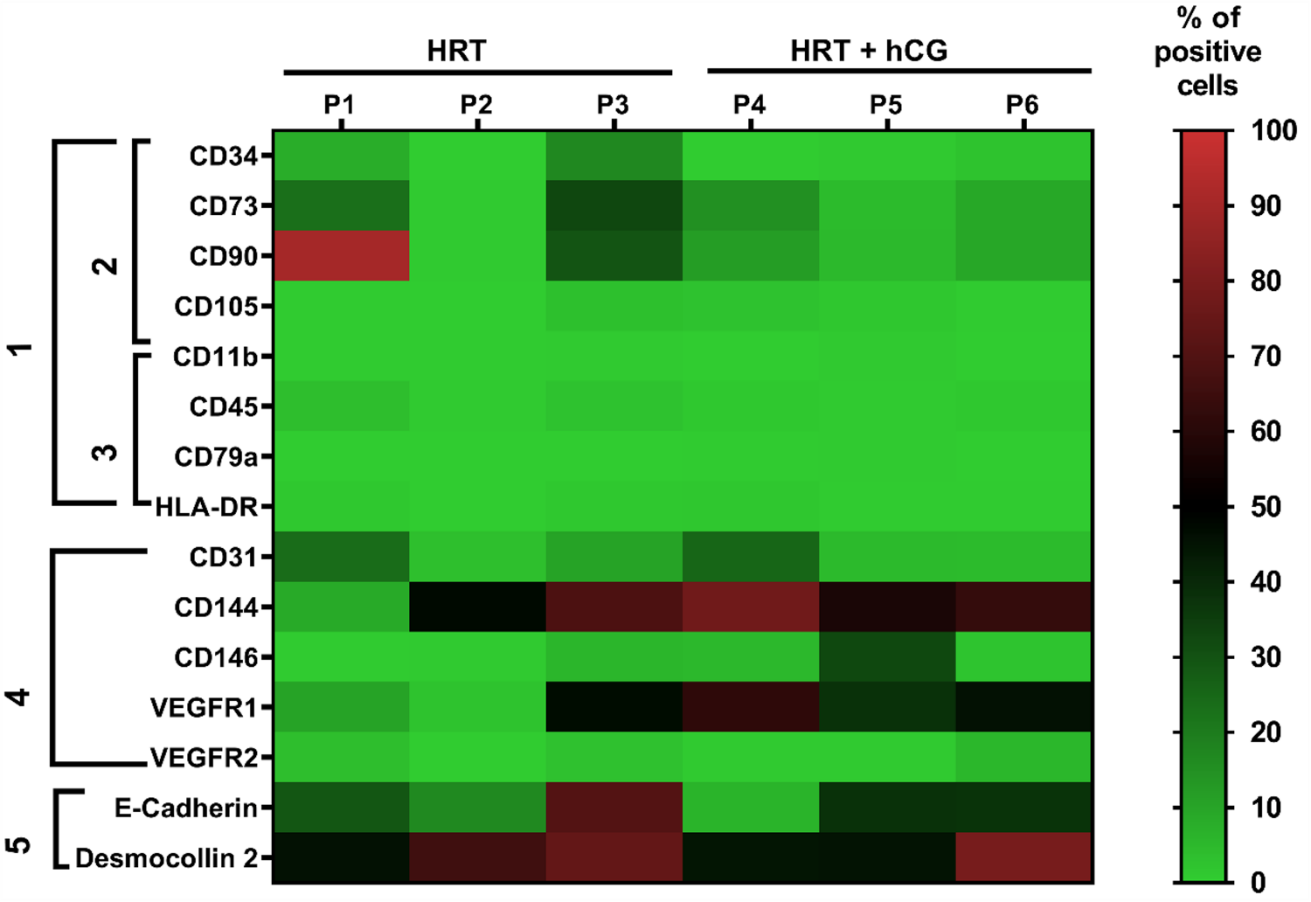
Protein expression in different cell clusters with and without hCG in human endometrium biopsies. Three patients per group were treated with hormonal replacement therapy (HRT) without hCG (HRT; P1–P3) and with hCG (HRT + hCG; P4–P6). The protein expression of the respective markers is shown with colors from light green (0% protein expression) to light red (100% protein expression). Here 100% corresponds to 20,000 cells. Cluster 1 corresponds to the marker set for MSC according to ISCT. Cluster 2 includes markers which are expressed on the stem and stromal cells and cluster 3 describes markers expressed on immune cells. Cluster 4 describes markers expressed on endothelial cells and cluster 5 markers expressed on epithelial cells

Six women in the HRT group were hormonally treated with micronized estradiol and vaginal progesterone only. Additionally, 6 women in the HRT + hCG group were hormonally treated with estradiol and progesterone plus intrauterine flushing with 1000 IU urinary hCG (Fig. 1; Table 1). There were neither significant differences in age between the two groups (HRT: 32.0 ± 4.8 years/HRT + hCG: 33.7 ± 2.7 years), nor body mass indices (BMI) (HRT: 26.9 ± 10.1 /HRT + hCG: 22.5 ± 4.3). Homeostasis model assessment indices (HOMA) were measured within two months before tissue sampling. Both groups show metabolic homogeneity as indicated by HOMA indices (HRT: 2.0 ± 0.3/HRT + hCG: 2.0 ± 0.5). Also, no difference existed in the time of infertility (initiation of diagnostics and ART treatment) in month (HRT: 16.2 month ± 4.5/HRT + hCG: 17.7 ± 5.9 month), in the number of previous embryo transfers (ETs, fresh and frozen) (HRT:  1.8± 0.9/HRT + hCG: 2.3 ± 1.0) or the cumulative number of embryos transferred (HRT: 3.5 ± 2.1/HRT + hCG: 4.3 ± 2.3). All women were nulliparous and had a first ICSI procedure before study enrollment. All patients in the HRT group had primary infertility. Likewise, four of the six patients in the HRT + hCG group. Two patients in the HRT + hCG group were secondarily infertile after early miscarriage (gestational week < 7) in their first pregnancy. In one patient of the HRT group, clinical inapparent endometriosis (ASRM grade I) was detected by laparoscopy in 2013. One in this group was treated with thyroxine 50, one woman in the HRT group had epilepsy treated with lamotrigine and a different one diabetes type 2 (DMT2) sufficiently controlled with an HbA1c of 6.2%. One woman in the HRT + hCG group had asthma without treatment > 3 months prior to study inclusion. One patient in the HRT + hCG group had a previous myomectomia in 2015 and one had Gilbert's syndrome. One woman had asthma and another had Hashimoto's thyroiditis which was treated with L-thyroxine 75. Other comorbidities or associated medications were not documented.

Screening of the endometrial tissue in the patients without (HRT group) and with hCG treatment (HRT + hCG group) provides a first overview (Fig. 1). Cellular populations were characterized with markers that are typical for the respective cellular cluster. ISCT markers which define an MSC (Fig. 1, cluster 1) were subdivided into two cellular clusters including markers typically expressed on stroma/stem cells (CD34, CD73, CD90 and CD105; Fig. 1, cluster 2) and immune cells (CD11b, CD45, CD79a, HLA-DR; Fig. 1, cluster 3). Endothelial cell–cell-contact molecules CD31 (platelet endothelial cell adhesion molecule; PECAM-1), CD144 (VE-Cadherin) and CD 146 (cell adhesion molecule S-Endo-1 antigen) were investigated in the endothelial cellular population together with VEGF receptors 1 and 2 (VEGFR1, VEGFR2) (Fig. 1, cluster 4). Epithelial cells were investigated in the epithelial cellular cluster by E-cadherin and desmocollin 2 expression (Fig. 1, cluster 5). The initial screening indicated that intrauterine hCG administration is more likely to alter the endothelial than the stroma and stem, immune or epithelial cells of the endometrium.

**Fig. 2:**
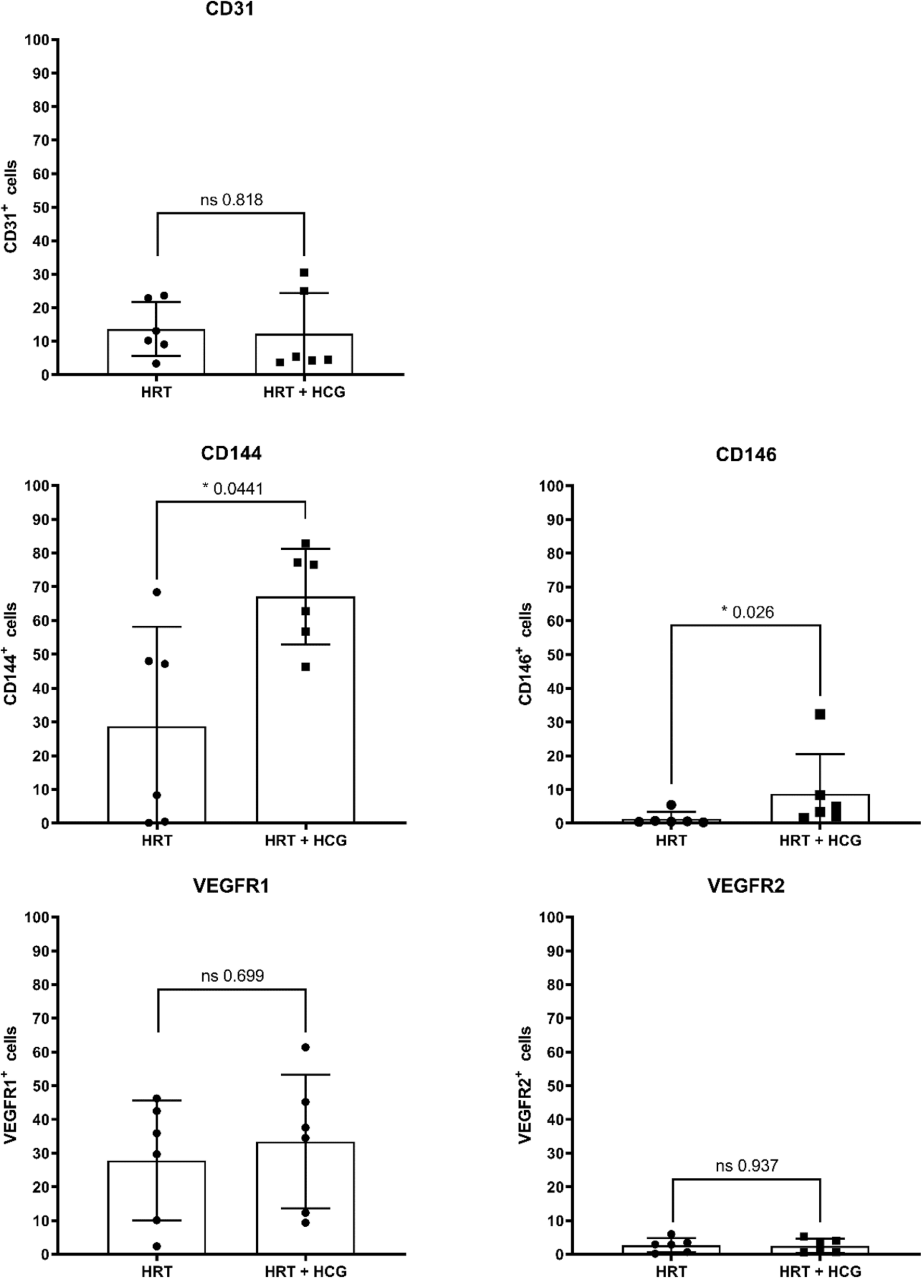
Flow cytometric characterization of the endothelial cell population in human endometrium with and without hCG treatment. Patients were treated with hormonal replacement therapy without hCG (HRT) and HRT with hCG (HRT + hCG). Protein expression of the respective markers is shown here in percent positive events, where 100% corresponds to 20,000 cells. Endothelial cell population is described by cell–cell adhesion molecules CD31, CD144, CD146 and the VEGFR-receptors VEGFR1 and VEGFR2 with *n* = 12

**Fig. 3:**
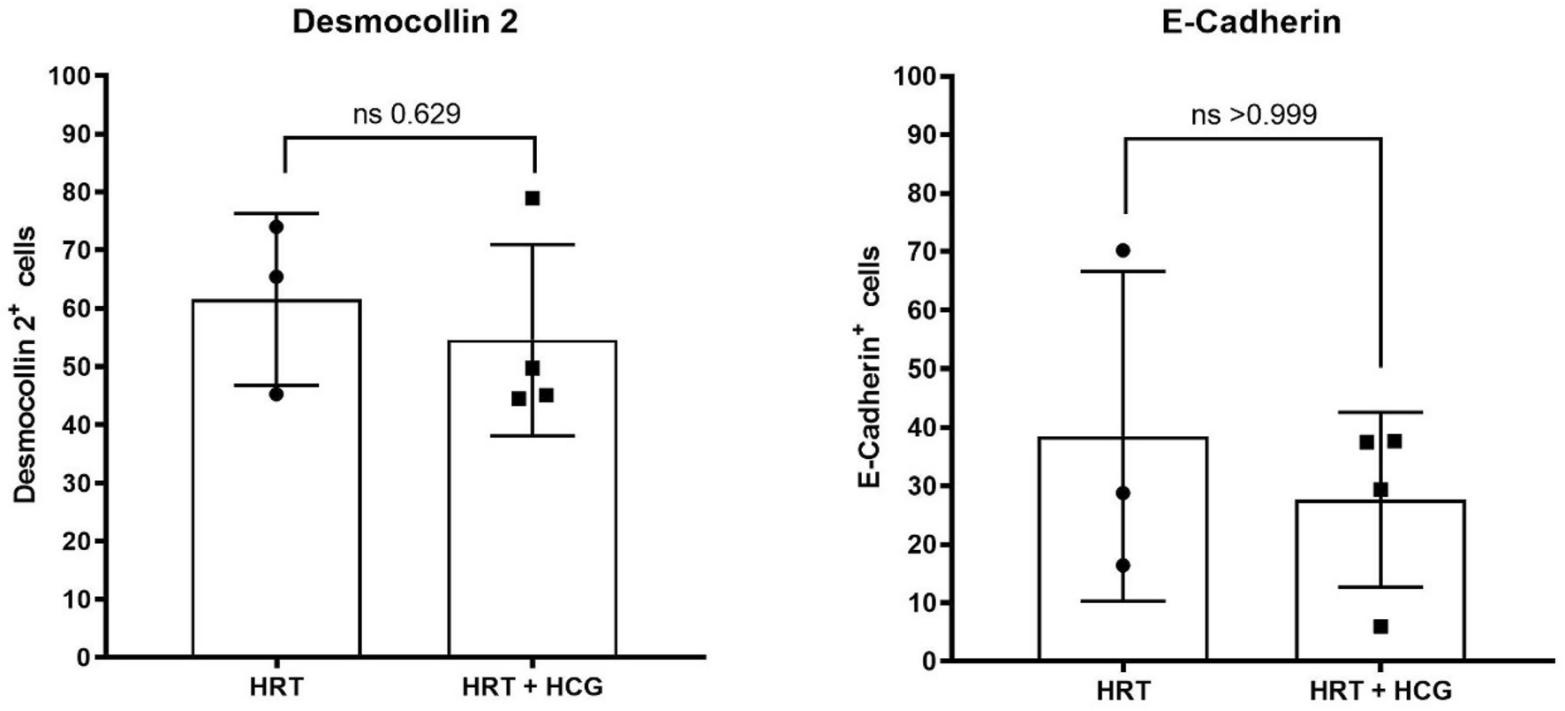
Flow cytometric characterization of the epithelial cell population in human endometrium with and without hCG treatment. Patients were treated with hormonal replacement therapy without hCG (HRT) and HRT with hCG (HRT + hCG). Protein expression of the respective markers is shown here in percent positive events, where 100% corresponds to 20,000 cells. Epithelial cell population is described by desmocollin 2 and E-cadherin with *n* = 7

**Fig. 4:**
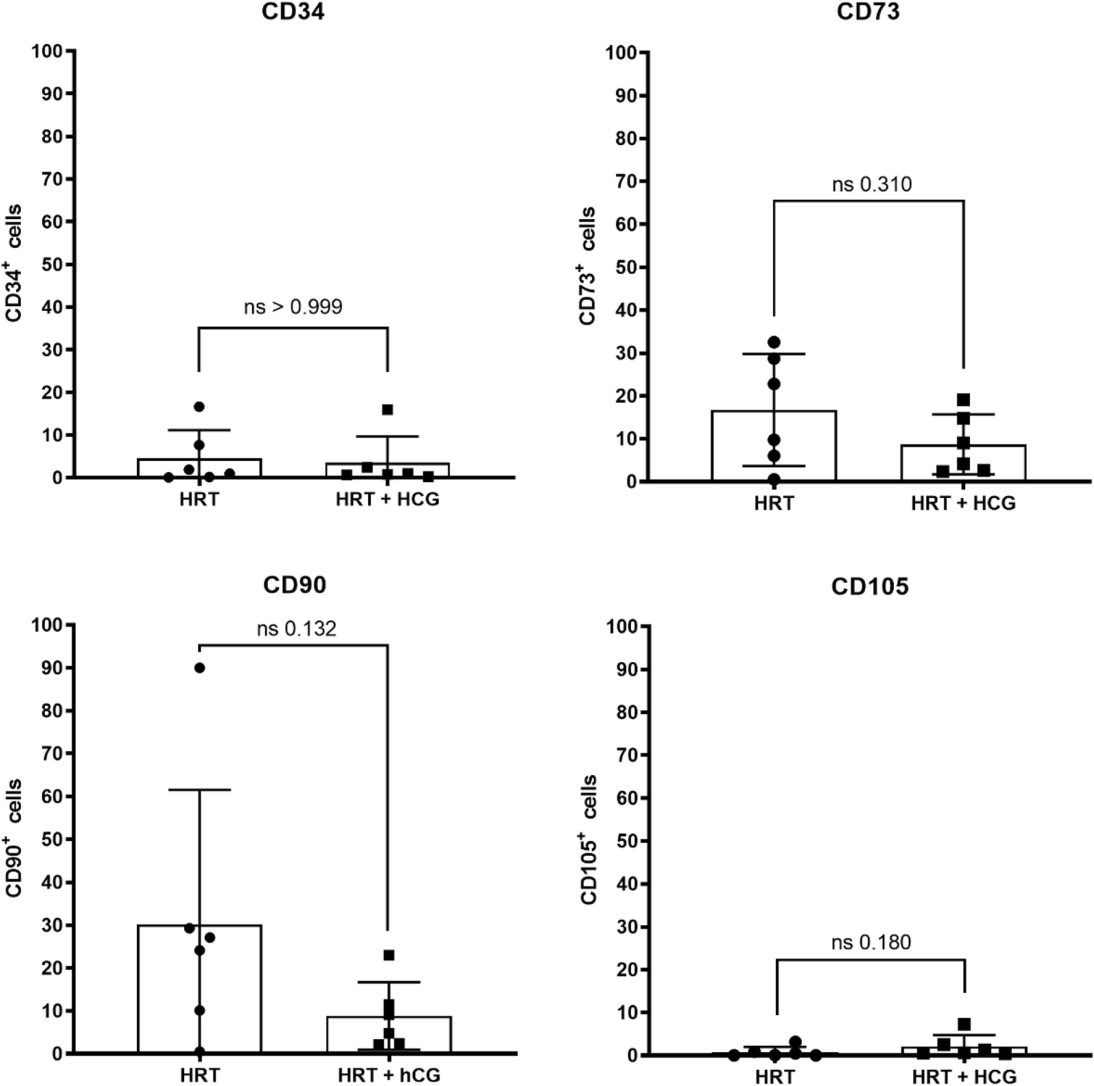
Flow cytometric characterization of the stroma/stem cell population in human endometrium with and without hCG treatment. Patients were treated with hormonal replacement therapy without hCG (HRT) and HRT with hCG (HRT + hCG). Protein expression of the respective markers is shown here in percent positive events, where 100% corresponds to 20,000 cells. Stroma/stem cell cluster described by the ISCT markers CD34, CD73, CD90 and CD105 with *n* = 12

**Fig. 5:**
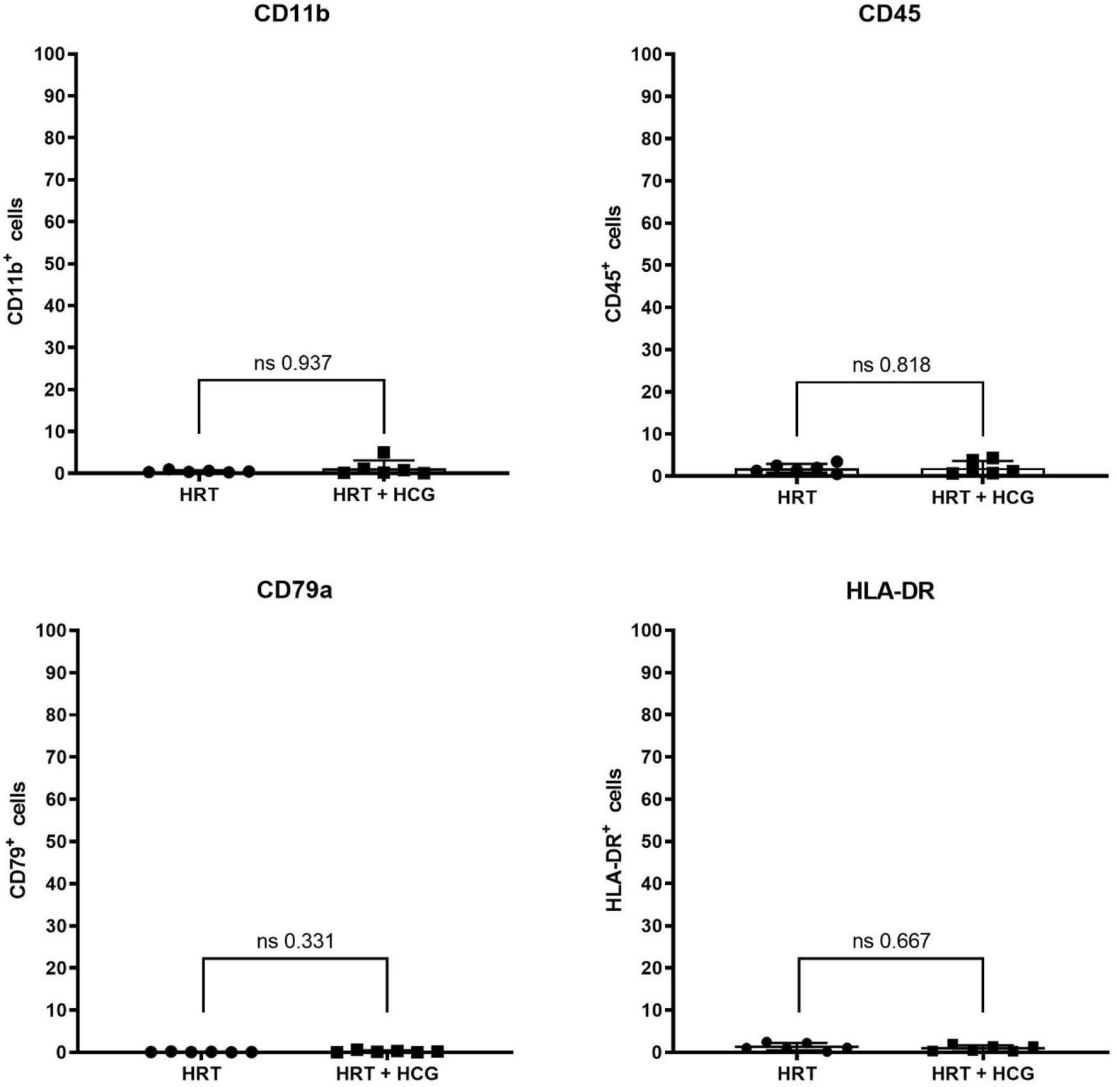
Flow cytometric characterization of the immune cell population in human endometrium with and without hCG treatment. Patients were treated with hormonal replacement therapy without hCG (HRT) and HRT with hCG (HRT + hCG). Protein expression of the respective markers is shown here in percent positive events, where 100% corresponds to 20,000 cells. Immune cell population is described by the ISCT markers described by CD11b, CD45, CD79a and HLA-DR with *n* = 12

Analysis of the complete collective of twelve women showed that intrauterine hCG administration significantly increased the proportion of CD144 and CD146-positive cells which are investigated in the endothelial cellular population as endothelial cell–cell contact molecules (Fig. 2). In the hCG-treated group the percentage of CD144-positive cells was significantly higher compared to the hCG-untreated group (HRT: 28.8 ± 29.4%/HRT + hCG: 63.7 ± 13.3%; *p* = 0.0411). In addition, in the hCG-treated group the percentage of CD146-positive cells was also significantly higher than in the hCG-untreated group (HRT: 1.3 ± 2.0%/HRT + hCG: 8.7 ± 11.8%; *p* = 0.0260). In contrast, the percentage of CD31-positive cells did not significantly increase or decrease by hCG administration (HRT: 13.7 ± 8.0%/HRT + hCG: 12.2 ± 12.2%; *p* = 0.8182). There was also no significant difference in the proportion of VEGR1- and VEGFR2-positive cells in the two groups, which were investigated for angiogenesis regulation (VEGFR1: HRT: 27.8 ± 17.8%/HRT + hCG: 33.4 ± 19.9%; *p* = 0.6991) (VEGF2: 2.7 ± 2.1%/HRT + hCG: 2.5 ± 2.0%; *p* = 0.9372) (Fig. 2)*.* Also, all other markers examined were not found to be significantly altered after hCG administration (Figs. 3, 4, 5). In the epithelial cell cluster, no significant difference was found between the treated and untreated groups (Fig. 3). Neither the epithelial cell–cell-contact molecule desmocollin 2 nor E-cadherin were expressed on less or more cells after hCG administration (desmocollin 2: HRT: 61.7 ± 14.8%/HRT + hCG: 54.6 ± 16.4%; *p* = 0.6286) (E-cadherin: HRT: 38.5 ± 28.2%/HRT + hCG: 27.7 ± 14.9%; *p* > 0.9999).

HCG administration did not significantly alter the percentage of cells positive for stroma/stem cell markers (Fig. 4). Percentage of cells expressing the stemness marker CD34 was not significantly altered by hCG administration (HRT: 4.5 ± 6.6%/HRT + hCG: 3.5 ± 6.1%; *p* > 0.999). As well percentage of CD73, CD90 and CD105-positve cells was not altered by hCG administration (CD73: HRT: 16.7 ± 13.1%/HRT + hCG: 8.7 ± 7.0%; *p* = 0.3095), (CD90: HRT: 30.2 ± 31.3%/HRT + hCG: 8.8 ± 7.9%; *p* = 0.1320). Markers of the immune cell cluster were also not affected by hCG administration (Fig. 5). Neither CD11b, CD45, CD79a nor HLA-DR were expressed on significantly more or less cells after hCG application (CD11b: HRT: 0.5 ± 0.3%/HRT + hCG: 1.3 ± 1.9%; *p* = 0.9372), (CD45: HRT: 1.9 ± 1.02%/HRT + hCG: 2.0 ± 1.6%; *p* = 0.8182), (CD79a: HRT: 0.1 ± 0.1%/HRT + hCG: 0.2 ± 0.2%; *p* = 0.3312) and (HLA-DR: HRT: 1.3 ± 0.9%/HRT + hCG: 0.9 ± 0.7%; *p* = 0.6667).

Multiple linear regression showed that 92.4% of the variance in the CD144 HRT + hCG group could be explained by age, BMI and HOMA (adjusted *R*^2^ = 0.924; Table 2). Age, but not BMI or HOMA, significantly influenced CD144 expression in the HRT + hCG group (p* = 0.031). In the CD144 HRT, CD146 HRT and in the CD146 HRT + hCG group, no significant effects of age, BMI or HOMA on the respective marker expression could be determined.

**Table 2:** Influence of age, BMI and HOMA on CD144 expression in the HRT + hCG group

Variable	Unstandardized regression coefficient	Standardized regression coefficient	Standard error
Constant CD144 HRT + hCG	− 202.100		
Age	7.128*	1.399*	1.287
BMI	− 0.157	− 0.046	0.919
HOMA	16.322	0.579	10.931
*R*^2^	0.969		
Adjusted *R*^2^	0.924		
*F* (*df* = 3;2)	21.154*		

*p < 0.05; **p < 0.01; ***p < 0.001

### Discussion

Implantation of the embryo into the female uterus is a finely orchestrated process involving multiple cell types, molecules and cytokines. The connection of the embryo to the maternal vasculature is a decisive process and essential for successful implantation, placentation and subsequent gestation [17]. This work investigates the role of pharmacological intrauterine flushing with 1000 IU urinary hCG in early secretory endometrium in vivo.

Following intrauterine hCG administration significantly more cells in the female endometrium expressed the endothelial cell adhesion molecules CD144 and CD146. CD144 is an endothelium-specific member of the cadherin family called VE-cadherin and belongs to the adherens junctions molecules [18]. CD146 is an endothelial cell–cell adhesion molecule as well expressed in the vascular system [19, 20]. All tree endothelial adhesion molecules investigated in here CD31, CD144, and CD146, regulate leukocyte trafficking and vascular permeability. In this work, it could be shown that significantly more cells expressed CD144 and CD146 after hCG administration, whereas CD31 expression was not significantly altered by hCG. Age, but not BMI and HOMA, had a significant effect on CD144 expression in the HRT + hCG group, whereas no significant effects of age, BMI, or HOMA on the respective marker expression could be determined in the CD144 HRT, CD146 HRT, and CD146 HRT + hCG groups. CD31 is positioned below the adherens junctions which consist among others of CD144 [20, 21]. CD146 is as well highly expressed at the endothelial cell–cell contacts [11] but did not colocalize with CD144 or CD31 [20]. A possible explanation to the observation that only the endothelial cell adhesion molecules CD144 and CD146 are expressed significantly different but not CD31 in the presence of hCG is that they are localized at different positions with respect to cell–cell contact sites. During hCG treatment, endothelial cell–cell contacts might have become more permeable and loose from an adluminal apical position to a basolateral location. Further analyses are certainly needed to support this idea. In this context, it would also be of great interest to investigate the effect of hCG on tight junctions, which are also located at endothelial cell–cell contact sites [18]. It was already shown that hCG can modulate the baboon endometrium by modulating the secretion profile of the epithelial glands during the WOI [6, 22]. Furthermore, in cell culture experiments hCG has been shown to up-regulate the formation of gap junctions in cells derived from term pregnancy human placenta [23]. Lei et al. demonstrated that human endometrial vascular smooth muscle and endothelium express human chorionic gonadotropin/human luteinizing hormone receptor (hCG/hLH) mRNA and suggested that hCG/hLH may directly regulate the blood flow in the human uterus [8]. Taken together, future studies should investigate whether hCG regulates vascular permeability in the endometrium via endothelial cell–cell contacts.

In this work, it was observed, that only some markers of the endothelial cluster were expressed differently after hCG administration implicating modulation of the endothelial cell–cell contacts in already existing vessels rather than supporting new vessel sprouting. VEGFR1 and 2, which actively and passively regulate vascularization, were not altered after hCG administration [20]. VEGFR2 is involved in endothelial cell proliferation, migration and vessel formation [24, 25]. VEGFR1 acts as a negative regulator of vessel formation since it binds soluble VEGF-A with a tenfold higher affinity than VEGFR2 [26, 27]. This could lead to the assumption that intrauterine hCG administration, alters existing vessels on the endothelial cell–cell contacts rather than the induction of new vessels formation.

Endometrial tissue is in a continuous remodeling mode with interindividual variations in time patterns and cellular subsystem composition. In our test paradigm, we tried to standardize endocrine conditions in this highly dynamic tissue by a strict hormonal endometrial preparation and biopsy protocol to create a homogeneous “endocrine environment” for human endometrium in vivo. The timely pattern of pharmacologic intervention and tissue sampling aims to simulate the clinical most promising procedure following meta-analysis [4]. Thus, hCG application to the endometrium was scheduled at the time of an average cleavage-stage embryo transfer to investigate early secretory endometrium around the time of embryo hatching and first trophoblast to endometrial luminal epithelia contact.

Clear limitations of our work are the small number of patients in our collective and the lack of clinical follow-up for pregnancy or birth rates. According to Green 1991, achievement of a statistical power of 0.8, where *β* = 0.2, *α* = 0.05 and *R*^2^ = 0.07, the sample size (*N*) must be at least 50 + 8 m, where *m* is the number of independent variables [28]. Thus, in our case *N* should be at least 74 subjects (*N* ≥ 50 + 8 × m; where *m* = 3; age, BMI, HOMA index). However, this is only true if the coefficient of determination *R*^2^ is of interest. For a determination of the influence of age, BMI and HOMA on CD marker expression, *N* have to be at least 107 according to Green (*N* ≥ 104 + *m*) [28]. Therefore, the results published here are best understood as an idea for a design of a follow-up study because it is imperative that the present work be verified in a larger collective and at best be embedded in ART treatment outcome measures.

However, the present work indicates that the application of urinary hCG preparations modulates endothelial cell–cell contacts, especially CD144 and CD146, in the human endometrium in vivo. The resulting clinical perspectives are that intrauterine hCG flushing might support aspects of endometrial vascularization. Following this hypothesis, most benefit of intrauterine hCG treatment could be reached for ART patients with an impaired vascularization process during endometrial tissue remodeling towards the window of implantation in frozen embryo transfer (fET) cycles. Further proof of this hypothesis in a larger study population could support a clinical treatment algorithm. Insufficient endometrial vascularity could be detected by endometrial testing in a first biopsy, followed by a second biopsy after intrauterine hCG application to verify improved endometrial remodeling towards the WOI before embryo-transfer.
